# Integrating Traditional Chinese Medicine and Nanotechnology for Enhanced Management of Anti-Tumor Drug Toxicity

**DOI:** 10.3390/molecules31152557

**Published:** 2026-07-23

**Authors:** Yueyao Tong, Keshu Sun, Jingbo Liu, Fengyun Li

**Affiliations:** 1 School of Chinese Materia Medica, Tianjin Key Laboratory of Therapeutic Substance of Traditional Chinese Medicine, Tianjin University of Traditional Chinese Medicine, Tianjin 301617, China; tongyy0711@163.com (Y.T.); 15937809781@163.com (K.S.); 2 College of Horticulture and Landscape Architecture, Tianjin Agricultural University, Tianjin 300384, China

**Keywords:** anti-tumor drugs, toxicity, traditional Chinese medicine, nanomedicines, pharmacological efficacy, synergy

## Abstract

While anti-tumor drugs markedly improve patient survival, dose-limiting toxicities remain major constraints on clinical efficacy and quality of life. Conventional management strategies lack timeliness and precision. Traditional Chinese medicine (TCM) and its active ingredients offer unique potential for mitigating anti-tumor drug toxicities through multi-component and multi-target regulation. However, the transformation of TCM is hampered by poor bioavailability and targeting. This review summarizes and evaluates an integrated strategy combining TCM with nanotechnology to develop novel nanomedicines. It elucidates the distinct toxicity mechanisms of chemotherapy drugs, targeted drugs, and immunotherapy drugs, revealing toxicopathological transitions from non-specific killing to microenvironment disruption and immune imbalance. Subsequently, it discusses the intervention mechanisms and research progress of TCM and its active ingredients targeting different categories of anti-tumor drug toxicity. To overcome delivery challenges, this review explores construction strategies for diverse nanodelivery systems, including carrier-free self-assembled nanomedicines, physically loaded nanomedicines, and chemically coupled nanomedicines, highlighting their value in organ-specific accumulation and controlled release. Finally, it objectively analyzes challenges in the clinical translation of these nanomedicines, encompassing safety and industrialization, while prospecting future trends, aiming to contribute to a new therapeutic paradigm focused on “toxicity attenuation and efficacy potentiation” and steer cancer treatment toward greater precision and intelligence.

## 1. Introduction

Since nitrogen mustard drugs were first used in clinics in the 1940s, anti-tumor drug therapy has been developing continuously. At the beginning of the 21st century, the widespread use of targeted drugs marked a new stage in tumor treatment. A variety of anti-tumor drugs emerged one after another and continued to evolve, significantly improving patients’ survival rates. However, their side effects are becoming increasingly prominent. Clinical data indicate that more than 80% of patients receiving targeted therapy will develop skin, heart, liver or kidney toxicity [1]. These adverse reactions frequently necessitate dose reduction or termination of treatment, which has become the main factor limiting clinical efficacy. Conventional management strategies, such as external use of urea ointment to treat skin toxicity or temporary drug interruption [2], are often hampered by delayed response, low specificity, and limited efficacy, posing significant clinical challenges and creating an urgent need for innovative solutions.

With its holistic, multi-component, and multi-target regulatory characteristics, TCM shows unique potential to alleviate the toxicity of anti-tumor drugs [3]. It can effectively target damaged cells or disordered metabolic pathways, alleviate toxic symptoms, and prevent treatment interruption. A key advantage of TCM lies in its ability to maintain or even enhance the anti-tumor efficacy of conventional drugs, epitomizing the concept of “differential toxicity regulation”, namely, selectively protecting normal tissues, while retaining or enhancing the killing effect on tumors. The newly proposed “vascular endothelial cell toxicity-initiated injury theory” further provides a new mechanism basis for targeted intervention of TCM [4].

However, TCM and its active ingredients often face challenges, such as poor lipid or water solubility, low stability, and a lack of targeting, which limit bioavailability and restrict clinical application. In recent years, the rapid development of nanotechnology has provided an effective way to solve the above problems [5]. Nano-delivery systems can improve the bioavailability of TCM and its active ingredients, facilitate their efficient crossing of biological barriers, and exert therapeutic effects at the target site [6]. Therefore, it is a promising strategy to combine the advantages of multi-target regulation in TCM with nanotechnology, and to realize accurate intervention through the “differential toxicity signaling map” under the guidance of the “vascular endothelial cell toxicity-initiated injury theory”. This strategy not only helps clarify the toxic mechanisms of anti-tumor drugs but also provides a new approach to reducing their toxic effects while maintaining anti-tumor efficacy. Based on this, this paper systematically summarizes the mechanisms of anti-tumor drug toxicity and discusses the potential of TCM and its active ingredients to alleviate drug toxicity and synergistically enhance anti-tumor effects. This study further puts forward an intervention scheme that combines the multi-target characteristics of TCM with the advantages of precise delivery of nanotechnology.

## 2. Classification and Toxicity Mechanism of Anti-Tumor Drugs

Although anti-tumor drugs have prolonged patient survival, their toxic effects have become key problems that limit their clinical application and patient quality of life [7]. At present, anti-tumor drugs commonly used in clinics are mainly divided into three categories: chemotherapy drugs, targeted drugs and immunotherapy drugs [8]. The mechanisms underlying their toxicity have evolved from nonspecific injury to an imbalance of precise regulation. Traditional chemotherapy mainly causes extensive cell damage, while targeted therapy may lead to dysfunction of specific pathways. The core challenge of immunotherapy lies in the overactivation of the immune system and its related adverse reactions.

Chemotherapy drugs induce immunogenic cell death (ICD), which broadly kills tumor cells by interfering with DNA synthesis, disrupting microtubule structure, or inhibiting topoisomerase. However, normal tissues such as bone marrow, gastrointestinal mucosa, and hair follicles also suffer severe damage [9]. Alkylating agents (such as nitrogen mustard), the first chemotherapy drugs, induce ICD through DNA alkylation, but their high reactivity causes extensive DNA damage in normal tissues, leading to bone marrow suppression and hepatotoxicity [10]. Platinum drugs (such as CDDP) form DNA-platinum adducts to impede replication. Their characteristic nephrotoxicity and neurotoxicity are mediated by drug accumulation via the copper transporter 1 (CTR1) in renal proximal tubule cells and dorsal root ganglia [11]. It is worth noting that the toxicity of chemotherapy drugs is dose-dependent, and their pharmacokinetic characteristics (such as plasma half-life and clearance rate) are closely related to tissue-specific toxicity. A classic example is the cardiotoxicity of DOX, which is directly related to its accumulation in mitochondria of myocardial cells and the generation of free radicals [12].In recent years, research on the toxic mechanisms of anti-tumor drugs has gradually deepened, from early single-cell killing to the understanding of systemic disorders of the tissue microenvironment. Taking paclitaxel (PTX) as an example, it not only causes cell cycle arrest by stabilizing microtubules, but more notably, its metabolites in vivo can activate the TLR4/NF-κB signaling pathway in vascular endothelial cells, promoting the release of proinflammatory cytokines and exacerbating microvascular injury and tissue toxicity [13]. This discovery reveals that chemotherapy drugs can affect the tumor microenvironment and systemic homeostasis through metabolic reprogramming, thereby extending their effects from direct cytotoxicity to microenvironment-mediated indirect toxicity. At the same time, different drugs have different toxic pathways due to their different mechanisms of action, underscoring the importance of multidimensional intervention at the level of cell killing and microenvironmental interactions.

Unlike traditional chemotherapy, although targeted drugs can provide precise treatment by inhibiting tumor-specific signaling pathways, their “off-target effects” can still cause damage to normal tissues. As the core hub of drug toxicity initiation [4], vascular endothelial cells play a special role in targeted therapy. For instance, VEGF inhibitors such as bevacizumab block the VEGF/VEGFR axis to suppress tumor angiogenesis, but they concurrently induce mitochondria-dependent apoptosis and autophagic flux blockade in endothelial cells, thereby compromising the integrity of the microvascular structure [14] and consequently triggering a cascade of organ-specific toxicities, including sinusoidal obstruction syndrome (SOS) and myocardial microcirculatory disorders [15]. Meanwhile, early warning signals of endothelial injury offer new insights into toxicity management. Studies have shown that the mitochondrial reactive oxygen species (mtROS) bursts and cytochrome c leakage caused by mitochondrial dysfunction can quickly activate the ASK1/JNK apoptosis-promoting pathway, forming an early-warning “self-amplification” signal cascade [16]. The bidirectional regulatory effect of targeted drugs further aggravates the complexity of toxicity. For example, EGFR inhibitors block the PI3K/Akt/FoxO3a pathway in tumor cells while aberrantly activating the NF-κB/COX-2 axis in myocardial microvascular endothelium, inducing an inflammatory cytokine storm and dose-limiting myocarditis [17]. Similarly, Tyrosine kinase inhibitors continue to activate the TGF-β/Smad pathway in fibroblasts while inhibiting tumor EGFR signaling, and drive renal fibrosis through alpha-smooth muscle actin (α-SMA) overexpression and collagen deposition [18]. Such irreversible pathological changes indicate that the balance between toxicity and efficacy depends on the integrated output of the signal networks within a specific microenvironment, rather than solely on the inhibition intensity of a single target.

Immunotherapy drugs achieve therapeutic effects by activating or restoring the body’s anti-tumor immune response, but their overactivation can lead to immune-related adverse events (irAEs), which may affect multiple organs, including the skin, intestines, liver, lungs, and endocrine system. The occurrence of irAEs is related to the bidirectional regulation of immune checkpoint inhibitors (ICIs) on T cell activation: on the one hand, ICIs block PD-1/PD-L1 or CTLA-4 pathways to release the inhibitory signal on T cells in the tumor microenvironment. On the other hand, they can break the peripheral immune tolerance, leading to the clonal expansion of autoreactive T cells and potentially triggering a cytokine storm. For example, CTLA-4 inhibitors, such as ipilimumab, enhance anti-tumor immunity by consuming regulatory T cells (Tregs), but simultaneously reduce the body’s tolerance to autoantigens, causing toxic reactions such as colitis and hypophysitis [19]. IrAEs caused by ICIs exhibit clear organ specificity, which is closely related to the immune microenvironment at the site of action. In the intestinal tract, the abundance of antigen-presenting cells and effector T cells in the intestinal mucosa allows anti-PD-1 ICIs to upregulate the expression of intestinal homing receptors, such as α4β7 integrin, promoting T cell migration to the intestinal mucosa and resulting in diarrhea and colitis. In the liver, ICIs disrupt the immune homeostasis maintained by high PD-L1 expression on Kupffer cells, leading to T cell-mediated hepatitis [20]. In addition, ICIs may induce systemic autoimmune diseases through cross-reactive antibodies or molecular simulation mechanisms. For instance, cross-reactivity between PD-1 inhibitors and thyroid peroxidase can further lead to hypothyroidism [21]. To sum up, the toxicity of immunotherapy drugs stems from their restructuring of immune homeostasis: while enhancing anti-tumor immunity, they often disrupt the balance of self-tolerance, leading to organ-specific immune overactivation and related damage.

Although the three categories of anti-tumor drugs differ in their targets and pathological characteristics, they share a common issue: all inevitably cause varying degrees of damage to normal tissues. This damage often involves cross-interference among multiple signaling pathways, which limits the efficacy of single-intervention strategies. Therefore, identifying safe strategies that can address these toxic reactions in a comprehensive manner has become an urgent priority. In this context, TCM and its active ingredients—many of which are derived from natural products—offer potential solutions for alleviating anti-tumor drug toxicity, owing to their diverse chemical structures and robust biological regulatory capabilities.

## 3. Targeted Intervention of TCM and Its Active Ingredients Against the Toxicity of Anti-Tumor Drugs

**Figure 1:**
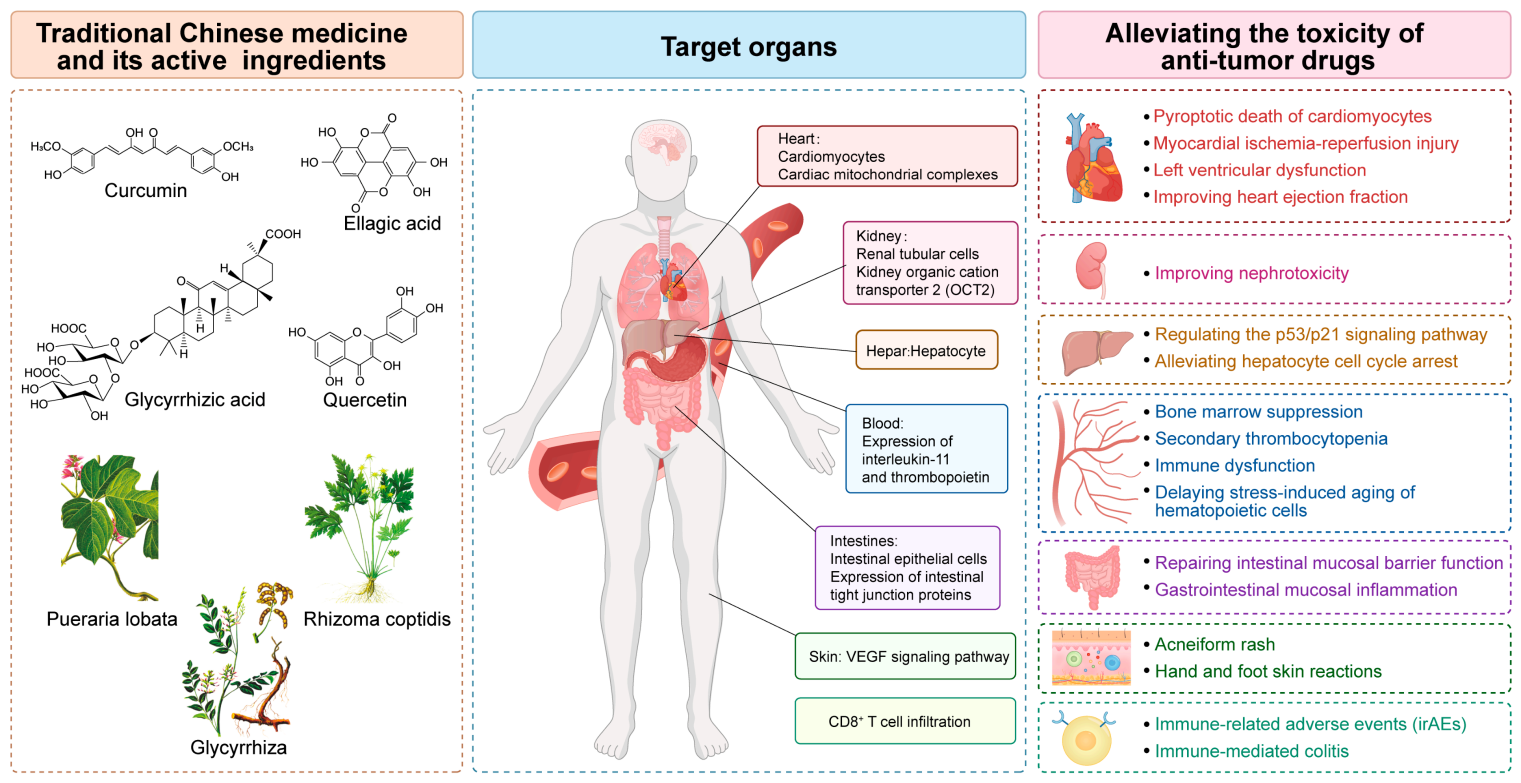
Schematic diagram of the intervention mechanisms of TCM and its active ingredients on organ toxicity.

There are significant differences in toxicity between different types of anti-tumor drugs, showing obvious class specificity. Against this background, TCM and its active ingredients have gradually demonstrated their potential in preventing and treating the toxicity of anti-tumor drugs, due to their unique advantages of multi-targeted action and low toxicity. Previous studies have shown that TCM can achieve targeted intervention in the toxicity of different types of anti-tumor drugs through various mechanisms, including antioxidant effects, anti-inflammatory actions, immune modulation and organ protection [22]. To systematically summarize the aforementioned intervention strategies and mechanisms of action, Table 1 outlines the toxicity types and underlying mechanisms for three classes of anti-tumor drugs—chemotherapy drugs, targeted drugs and immunotherapy drugs—along with the corresponding detoxification mechanisms and key effects of active ingredients and compound formulations in TCM. It clearly illustrates the “toxicity–detoxification” correspondence, providing an intuitive reference framework for subsequent mechanistic studies and clinical translation. The following will systematically expound the latest research progress in TCM targeted intervention of specific toxicities caused by three major categories of anti-tumor drugs and deeply discuss the mechanisms of action and clinical transformation potential of TCM. Figure 1 systematically illustrates the intervention mechanisms and strategic frameworks of various TCM and its active ingredients, which act on different target organs, thereby reducing adverse reactions caused by different types of anti-tumor drugs.

### 3.1. Intervention Mechanisms and Strategies for the Toxicity of Chemotherapy Drugs

Chemotherapy drugs (such as platinum-based drugs, anthracycline drugs, and antimetabolite drugs) play an anti-tumor role by nonspecifically killing rapidly proliferating cells and often cause dose-limiting adverse reactions, such as bone marrow suppression, cardiotoxicity, liver injury, nephrotoxicity, and gastrointestinal mucositis. The following sections systematically expound on the intervention mechanisms and strategies of TCM and its active ingredients from three aspects: organ toxicity, blood system toxicity, and gastrointestinal toxicity.

In terms of organ toxicity, chemotherapy drugs, because of their lack of specific targeting ability, not only kill rapidly proliferating cancer cells, but also attack normal cells that divide rapidly in the human body, and lead to functional damage to important organs in the body through various mechanisms, such as inducing oxidative stress and apoptosis. Studies have shown that curcumin, an active ingredient of TCM, can inhibit the activity of the NLRP3 inflammasome related to pyroptosis by activating the PI3K/Akt/mTOR signaling pathway, thus effectively alleviating myocardial pyroptotic death induced by DOX and cardiac dysfunction [23]. In addition, silymarin can significantly alleviate DOX-induced cardiotoxicity through multiple mechanisms, including antioxidation, anti-inflammation, and anti-apoptosis. Clinical studies have further demonstrated that silymarin can improve cardiac function indices, such as cardiac ejection fraction, in patients undergoing anthracycline chemotherapy [24]. Meanwhile, protecting liver and kidney function is also a core aspect in the management of anti-tumor drug toxicity. Taking quercetin as an example, it can reduce the accumulation of CDDP in renal tubular cells by inhibiting the function of organic cation transporter 2 (OCT2) in the kidney, and activate the Nrf2/ARE signaling pathway to enhance the activity of the antioxidant enzyme system, thus significantly alleviating the nephrotoxicity of CDDP [25]. Huaiqi huang granules effectively mitigate DOX-induced nephrotoxicity by significantly enhancing the expression of nephrin, a key podocyte slit diaphragm protein, and suppressing the overactivation of the NF-κB inflammatory signaling pathway [26]. The above research shows that TCM and its active ingredients can protect against organ toxicity in multiple ways by regulating oxidative stress, inflammatory responses, and apoptosis.

In the blood, chemotherapy drugs will indiscriminately attack all actively dividing cells, while hematopoietic stem and progenitor cells in bone marrow proliferate vigorously; as a result, these drugs directly induce apoptosis of these cells and deplete the precursors of blood cells in various lineages, leading to acute bone marrow suppression. In addition, chemotherapy drugs will also destroy the hematopoietic microenvironment of bone marrow, interfere with the normal hematopoietic signaling pathway, and further aggravate the decline in the number of white blood cells, platelets and red blood cells in peripheral blood by triggering the aging of hematopoietic stem cells, thus causing clinical complications such as infection, anemia and bleeding. In terms of TCM intervention, the aqueous extract of carica papaya can effectively alleviate the bone marrow suppression and secondary thrombocytopenia induced by carboplatin chemotherapy by significantly upregulating the mRNA expression level of IL-11 and synergistically regulating the expression of thrombopoietin [27]. Ganoderma lucidum polysaccharides can increase the production of short-chain fatty acids (SCFAs) in the intestine by regulating the intestinal flora-immune axis, thereby promoting the expression of granulocyte colony-stimulating factor (G-CSF) and the survival and differentiation of hematopoietic progenitor cells, and ultimately alleviating bone marrow suppression and immune dysfunction caused by 5-FU [28]. In addition, angelica sinensis polysaccharides can delay the stress aging of hematopoietic cells by improving the redox balance of bone marrow stromal cells [45]. Ganoderma lucidum spores protect hematopoietic function through antioxidation and anti-apoptosis [46]. These ingredients not only directly act on hematopoietic stem cells but also indirectly relieve bone marrow suppression through immune regulation and intestinal flora intervention, thereby embodying the therapeutic characteristics of multi-target and holistic regulation in TCM.

In terms of gastrointestinal toxicity, chemotherapy drugs directly damage metabolically active gastrointestinal mucosal cells, inhibit their normal repair and proliferation, and cause mucosal barrier damage, inflammation, and even ulcers, thereby triggering a series of symptoms. In addition, these drugs will interfere with the neuroregulatory function of the intestine and disrupt the balance of intestinal flora, resulting in gastrointestinal peristalsis rhythm disorder and dysfunction of absorption and secretion, which is manifested as diarrhea or constipation. In view of the above-mentioned toxic reactions, many ingredients of TCM show various protective mechanisms. Curcumin can significantly reduce the levels of proinflammatory factors in an intestinal mucosal injury model induced by 5-FU chemotherapy by inhibiting the IL-6/STAT3 signaling pathway and improving intestinal permeability, thereby repairing intestinal mucosal barrier function [29]. Ginsenoside Rh4 can effectively alleviate irinotecan-induced gastrointestinal mucositis by enriching beneficial bacteria, restoring the expression of intestinal tight junction proteins, and inhibiting the TLR4/NF-κB inflammatory pathway [30]. The protective effect of hesperidin focuses on repairing the physical barrier, directly strengthening the junction structure between intestinal epithelial cells by specifically upregulating the expression of zonula occludens-1 (ZO-1), claudin-1, and other tight junction proteins, and it has a clear improvement effect on repairing barrier damage caused by irinotecan and other drugs [31]. In a multicenter phase III randomized placebo-controlled clinical trial, the JianPi-BuShen formula significantly increased the adjuvant chemotherapy completion rate from 47.6% in the placebo group to 63.0% in the treatment group among 375 patients with stage II–III colon cancer after radical resection by effectively alleviating chemotherapy-induced toxicities, including gastrointestinal symptoms (nausea and vomiting) and myelosuppression, associated with oxaliplatin plus capecitabine [32]. As shown in the above cases, TCM and its active ingredients specifically intervene in the gastrointestinal toxicity induced by chemotherapy drugs through a multi-target and multi-pathway approach, embodying the therapeutic characteristics of TCM, which involve overall regulation, toxicity attenuation and efficacy potentiation.

### 3.2. Intervention Mechanisms and Strategies for the Toxicity of Targeted Drugs

Targeted drugs (such as EGFR inhibitors, VEGF inhibitors, and TKI) can specifically target tumor cell signaling pathways to exert therapeutic effects, but they are often associated with adverse effects such as hypertension and proteinuria. The following systematically expounds their manifestations in organ toxicity, gastrointestinal toxicity and skin toxicity and the intervention mechanisms of TCM and its active ingredients.

Among organ toxicities, cardiotoxicity is most common with HER2 inhibitors and anti-angiogenic drugs, which can lead to left ventricular dysfunction and hypertension. Studies have shown that salvianolic acid B has a clear protective effect on myocardial ischemia–reperfusion injury caused by sunitinib and trastuzumab [33]. It can restore the normal function of the eNOS/cGMP signaling pathway by inhibiting endothelial injury mediated by ROS, thereby improving vasodilation and tissue perfusion and alleviating cardiac dysfunction. Bevacizumab can inhibit mitochondrial complex II activity and disrupt energy metabolism, thereby causing cardiotoxicity. However, ellagic acid, an active ingredient of TCM, can effectively reduce mitochondrial oxidative stress damage and restore electron transport chain function through its strong antioxidant properties, thereby exerting a clear protective effect against left ventricular dysfunction caused by bevacizumab [34]. Hepatotoxicity is mostly caused by TKI such as imatinib and sunitinib, which are characterized by increased transaminase levels and liver cell damage. In terms of the intervention strategies of TCM and its active ingredients, glycyrrhizic acid can reduce gefitinib-induced cell cycle arrest by regulating the p53/p21 signaling pathway, thereby protecting liver function [35]. These studies suggest that the ingredients of TCM can mitigate organ toxicity induced by targeted drugs through multiple mechanisms, such as antioxidation and anti-inflammation, and exhibit systemic regulatory effects.

In terms of gastrointestinal toxicity, targeted drugs can directly cause mucosal inflammation, ulcers, and damage intestinal barrier function by inhibiting the specific signaling pathways required for the growth and repair of gastrointestinal mucosal cells, thus leading to common symptoms such as diarrhea. Ruscogenin, an active ingredient from ophiopogon japonicus, can effectively alleviate the intestinal barrier dysfunction caused by dasatinib, an anti-tumor drug, through a dual pathway mechanism: on the one hand, it activates the ErbB4/YAP signaling pathway to promote the expression of connexin, on the other hand, it inhibits the ROCK/MLC signaling pathway to reduce the phosphorylation of myosin light chain, thus stabilizing the intestinal vascular endothelial barrier and reducing permeability [36]. At the same time, the intervention of YiQiFuMai powder injection (YQFM) has also been shown to inhibit the ROCK/MLC signaling pathway, upregulate the expression of tight junction proteins, and reduce vascular permeability, thus alleviating intestinal leakage caused by dasatinib [37]. In a randomized double-blind placebo-controlled trial involving 320 patients with metastatic colorectal cancer, Huangci Granule combined with chemotherapy and cetuximab or bevacizumab significantly prolonged median progression-free survival and improved quality-of-life indicators including role functioning, social functioning, fatigue, and appetite loss, with *p* < 0.05. Furthermore, the incidence rates of grade 3 to 4 treatment-related adverse events such as leukopenia, fatigue, appetite loss, and hypertension were significantly reduced. These findings indicate that TCM can alleviate targeted drug toxicity through synergistic mechanisms, thereby providing high-level evidence-based support and a clinical translational paradigm for precise interventions that integrate Chinese and Western medicine [38].

Skin toxicity induced by targeted drugs is also a common adverse reaction. Drugs such as gefitinib and erlotinib often cause acne-like rashes and dry skin. In the intervention strategy, honeysuckle therapy can significantly reduce the severity and incidence of acne-like rash caused by EGFR inhibitors by inhibiting key inflammatory signaling pathways, such as MAPK and TNF, through its core active ingredients, and by regulating EGFR-related targets. Clinical controlled study results showed that adjuvant treatment with honeysuckle reduced the incidence of rash from 71.7% in the conventional treatment group to 56.5%, decreased the proportion of severe rash of grade 3 or above from 21% to 10%, and shortened the median time to pruritus relief from 58 days to 22 days [39]. Furthermore, a study found that the topical application of the Chinese herbal formula shouzuping, combined with urea ointment, effectively alleviated severe hand-foot skin reaction (HFSR) induced by regorafenib. The herbal ingredients work by modulating local VEGF signaling, inhibiting the release of inflammatory factors, and improving microcirculation and repair capacity in damaged skin, thereby reducing symptoms such as pain, blisters, and hyperkeratosis on the hands and feet of patients [40].

### 3.3. Intervention Mechanisms and Strategies for the Toxicity of Immunotherapy Drugs

Immune checkpoint inhibitors (such as PD-1/PD-L1 inhibitors and CTLA-4 inhibitors) have achieved remarkable effects in the treatment of various malignant tumors by blocking immunosuppressive signals and activating the anti-tumor immune response mediated by T cells. However, this overactivation of the immune system may also lead to an imbalance in immune tolerance, which in turn may result in immune-related adverse events (irAEs) across multiple systems and organs.

Immune checkpoint inhibitors often cause irAEs with excessive inflammatory reaction and T cell dysfunction as the main manifestations. Compound prescriptions of TCM show intervention potential through a multi-target mechanism. For example, Gegen Qinlian Decoction, mainly composed of pueraria lobata, rhizoma coptidis, radix scutellariae and glycyrrhiza, was used to intervene patients with non-small cell lung cancer (NSCLC), and it was found that it can alleviate the immune overactivation and related organ toxicity caused by PD-1/PD-L1 inhibitors by inhibiting pyroptosis and the release of related inflammatory factors mediated by the NLRP3 inflammasome, which embodies the characteristics of the compound in clearing away heat and toxic materials and regulating immune homeostasis [41]. On the other hand, curcumin has been demonstrated in head and neck cancer models to downregulate the expression of immunosuppressive ligands, such as PD-L1, PD-L2, and galectin-9, on tumor cell surfaces, while blocking multiple immune checkpoint signals, including PD-1 and TIM-3 on T cells. Thereby, it restores the function of CD8^+^ T cells and mitigates immunotherapy-related T cell exhaustion toxicity [42].

Immune colitis is the most common gastrointestinal immune-related adverse event (irAE) associated with immune checkpoint inhibitors, and its clinical management is particularly important. Among these, CTLA-4 inhibitors are more likely to cause severe diarrhea and colon inflammation. Regarding this toxicity, studies have found that various TCM, such as kushen decoction, baicalin, and epimedium, can alleviate inflammatory reactions by regulating the balance of Th17/Treg cells, inhibiting pro-inflammatory factors (such as IL-6 and IL-17), and enhancing anti-inflammatory factors (such as IL-10 and TGF-β) through immune regulatory pathways, as well as repairing intestinal barrier function and regulating intestinal flora, thereby treating colitis induced by immune checkpoint inhibitors [43]. In addition, as the active ingredient of atractylodes macrocephala, atractylenolide II can enhance the infiltration of CD8^+^ T cells and inhibit the inflammatory response by blocking the NF-κB p65/PD-L1 signaling pathway in tumor cells, and enhance the anti-tumor efficacy in cooperation with PD-1 inhibitors [44], significantly reducing the incidence of immune-related colitis [47]. It reflects the dual regulatory advantages of “toxicity attenuation and efficacy potentiation”.

By modulating the tumor microenvironment, gut microbiota, and immune cell function, TCM achieves bidirectional regulation of immune checkpoint inhibitor-related toxicity, thereby enhancing therapeutic efficacy while preventing or alleviating immune-related adverse events. Clinical and experimental studies have shown that Gegen Qinlian Decoction reduced the incidence of irAEs from 59.57% to 31.91% and extended the median time to onset from 8.7 weeks to 14.9 weeks [41]; Qigui Yishen Decoction reduced blood urea nitrogen levels from 8.59 to 4.03 mmol/L and serum creatinine levels from 150.59 to 60.03 μmol/L in patients with immune-mediated acute kidney injury, and increased the estimated glomerular filtration rate to 60.03 mL/min; the Yifei Decoction combined with corticosteroids reduced the severity score of immune-mediated pneumonia to 1.63; and external TCM treatments reduced the incidence of skin adverse reactions from 58.33% to 29.17% [48]. In addition, several registered clinical trials are currently underway, covering interventions for ICIs-related toxicity using TCM therapies such as electroacupuncture and Piyan Ning. These advances indicate that, leveraging its multi-target immunomodulatory properties, TCM can not only effectively prevent and alleviate ICIs-related multi-organ toxicity but also demonstrate significant translational medical value, providing an evidence-based medical pathway with Chinese characteristics for improving immunotherapy support regimens.

Based on the research progress in toxicity intervention for the aforementioned three classes of anti-tumor drugs, TCM and its active ingredients, owing to their multi-target, multi-pathway, and holistic regulatory properties, have demonstrated significant potential in regulating key processes such as oxidative stress, inflammatory responses, apoptosis, and immune homeostasis. However, the clinical application of active components of TCM still faces several inherent limitations that require objective scrutiny. On the one hand, most TCM and its active ingredients share common issues such as poor water solubility, low metabolic stability, lack of organ-specific targeting, and rapid clearance from the body, leading to insufficient bioavailability. On the other hand, some TCM formulations themselves pose potential toxicity risks. Studies have shown that certain alkaloid ingredients in TCM can induce various adverse reactions, including hepatotoxicity, nephrotoxicity, neurotoxicity, and cardiotoxicity. Due to their lack of targeting ability, they may produce nonspecific accumulation and effects in normal tissues. Therefore, it is particularly urgent to address these limitations through modern scientific and technological advancements. In recent years, nanomedicine delivery systems have offered promising solutions to overcome these challenges. By controlling the release rate and pathway of drugs, the active ingredients can be specifically accumulated and released at the tumor site, thereby reducing their distribution in normal tissues. While improving the solubility of TCM and prolonging their circulation time in the body, this effectively reduces their nonspecific effects on normal tissues. These advantages make nanotechnology promising for further enhancing the clinical translational value of toxicity management in TCM.

## 4. Application and Challenges of Nanotechnology in the Intervention of Anti-Tumor Drug Toxicity

Although TCM and its active ingredients can effectively alleviate the dose-limiting toxicity caused by anti-tumor drugs in many ways, their clinical application has long been limited by inherent limitations, resulting in insufficient bioavailability. The emergence of nanotechnology offers an innovative approach to solving the above problems. By converting drugs into nanomaterials or loading them into nanocarriers, their solubility can be significantly improved and their circulation time prolonged, thereby enhancing the efficacy of TCM in reducing the toxicity of anti-tumor drugs. Furthermore, nanocarriers can co-load the ingredients of TCM and anti-tumor drugs and coordinate their pharmacokinetic behaviors through their controlled-release characteristics, thereby reducing systemic toxicity while enhancing anti-tumor efficacy. This multi-dimensional strategy not only addresses the delivery efficiency of TCM, but also provides a new paradigm for achieving precise attenuation in anti-tumor treatment.

### 4.1. Types and Characteristics of Nanomedicines

**Figure 2:**
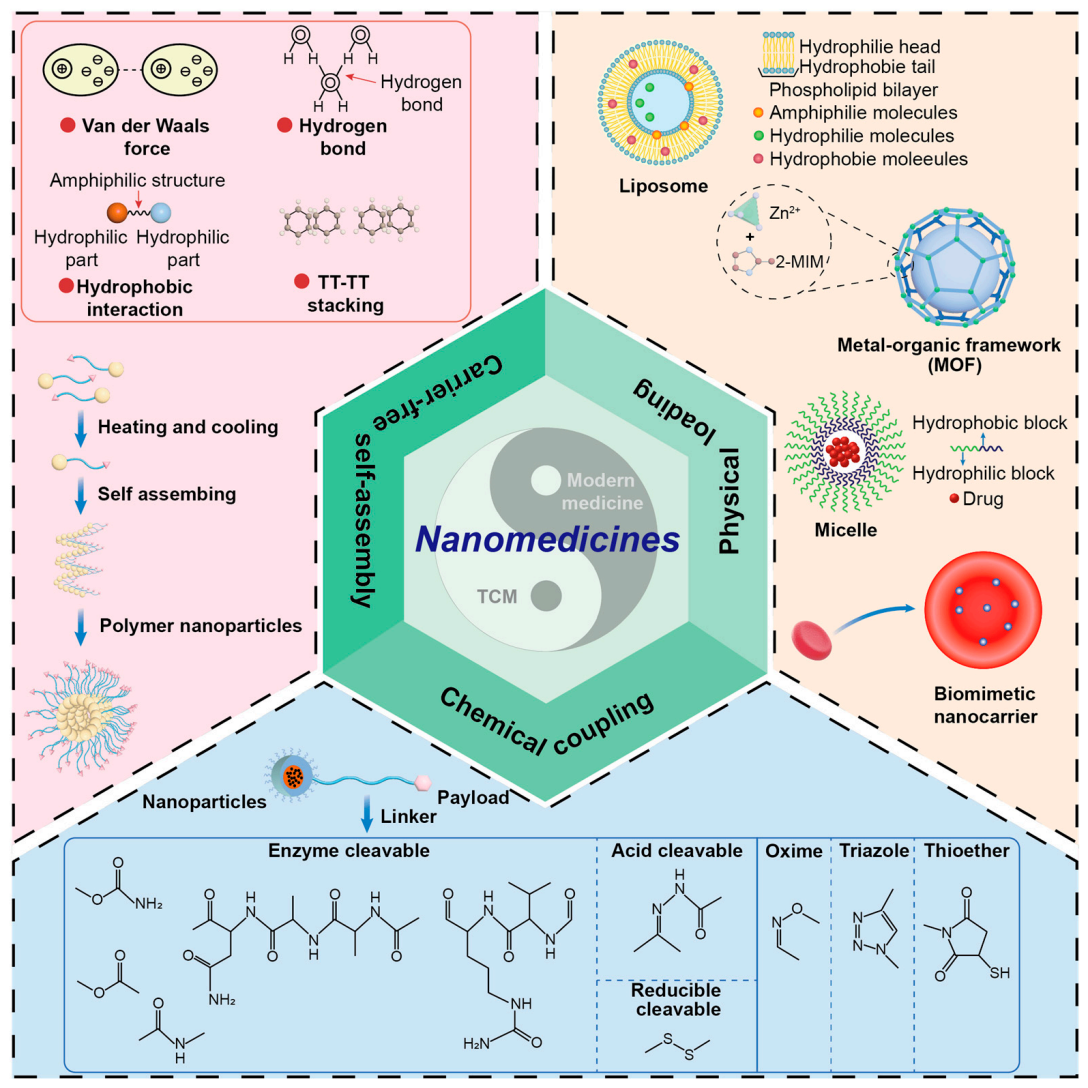
Summary diagram of types and characteristics of nanomedicines.

As a key strategy to improve the attenuation efficiency of TCM, nanomedicines are mainly constructed in three forms: carrier-free self-assembled nanomedicines, physically loaded nanomedicines, and chemically coupled nanomedicines via covalent bonding. By virtue of their unique effects, these nano-systems can significantly enhance the dispersion and dissolution rate of insoluble ingredients of TCM, and extend their circulation time in the blood by regulating the physical and chemical properties of carriers, such as surface modification and size regulation, thus effectively overcoming the bottleneck of low bioavailability of TCM preparations, achieving the enrichment of drugs in target organs, and laying the foundation for accurately reducing the specific organ toxicity of anti-tumor drugs. Figure 2 provides a systematic overview of the current mainstream types of nanomedicines and their assembly mechanisms, and these mechanisms include strategies such as hydrophobic interactions and hydrogen bonding-driven phospholipid bilayer formation in amphiphilic molecules, self-assembly of block copolymers, coordination and complexation of metal ions with organic ligands, controlled cleavage of chemical linkages in response to stimuli, and thermally induced self-assembly. This provides a structural basis for further discussion of the application of nanomedicines in intervening in the toxicity mechanisms of anti-tumor drugs.

#### 4.1.1. Carrier-Free Self-Assembled Nanomedicines

Carrier-free self-assembled nanomedicines are the spontaneous aggregation of active ingredients from TCM formulas into structurally ordered nano-assemblies through non-covalent forces such as hydrogen bonding, π-π stacking, hydrophobic interactions, and electrostatic interactions, triggered by physical methods like heating and cooling, without requiring exogenous carrier materials [49]. The formation method for this type of nanomedicine is simple and environmentally friendly. For example, through solvent exchange, heating and cooling, or direct water-phase self-assembly, TCM can self-organize into nanoparticles, nanofibers, or micelles under specific conditions, which exhibit good biocompatibility and biodegradability. For example, berberine can readily form butterfly shaped one-dimensional units with cinnamic acid via hydrogen bonding and π-π stacking, owing to its multi-aromatic ring structure and quaternary ammonium ion characteristics, and then assemble into three-dimensional nanoparticles [50]. This self-assembly process enables poorly soluble active components of traditional Chinese medicine to be effectively encapsulated or embedded within nanostructures. Leveraging the excellent drug-loading capacity and superior biocompatibility of nanomedicines, this approach can significantly improve the solubility of these components, prolong their circulation time in the body, and confer passive targeting capability. At the same time, this kind of nanomedicine has outstanding performance in alleviating the toxicity of anti-tumor drugs. For example, in the baicalin peptide conjugate (BA-FFYEEG-ARVYIHPF), the phenylalanine-tyrosine (FFY) motif provides the driving force for self-assembly, while the ARVYIHPF peptide sequence specifically targets the angiotensin II type 1 receptor (AT1R), which is upregulated after myocardial injury. The introduced glutamic acid (E) residues enhance overall hydrophilicity to stabilize the assembled structure. Triggered by heating-cooling cycles, this amphiphilic molecule undergoes a conformational transition and arranges itself in an orderly manner to form helical nanofibers with a poly-L-proline type II extended helical structure. Functionally, this self-assembled system not only greatly improves the water solubility and stability of poorly soluble baicalin, but more importantly, enables precise recognition of injured cardiomyocytes through the active targeting effect of the ARVYIHPF peptide, thereby achieving efficient delivery of baicalin to the cardiac region. Compared with free baicalin, AT1R-targeted baicalin delivery exhibits superior cardiac accumulation and better therapeutic outcomes in a mouse model of doxorubicin-induced cardiomyopathy [51].

Generally, carrier-free, self-assembled nanomedicines of TCM form a targeting system through molecular self-assembly, which not only simplifies the preparation process and avoids carrier-related toxicity but also enhances the solubility and stability of TCM, thereby achieving synergistic attenuation and providing innovative ideas for the toxicity management of anti-tumor drugs.

#### 4.1.2. Physically Loaded Nanomedicines

Physically loaded nanomedicines provide a systematic solution to overcoming the bottlenecks of low bioavailability and poor targeting by introducing functional materials to encapsulate active ingredients of TCM. Among these carriers, liposomes, as one of the most promising carriers for clinical translation, can be prepared by thin-film hydration, ethanol injection, reverse-phase evaporation, and high-pressure homogenization. Liposomes are typically composed of amphiphilic molecules such as phospholipids and surfactants. Phospholipid molecules spontaneously assemble in the aqueous phase to form closed vesicular structures, encapsulating hydrophilic drugs in the internal aqueous phase or embedding hydrophobic drugs into the lipid bilayer. Liposomes can significantly enhance the solubility and stability of poorly soluble ingredients of TCM, slow down the drug release rate, prolong the circulation time in vivo, and alter the tissue distribution profile of drugs, thereby improving oral absorption efficiency. As an example, baicalin liposomes prepared by effervescent dispersion technology increased the oral bioavailability of baicalin to three times that of the carboxymethylcellulose sodium suspension, and the peak plasma concentration reached 2.82 times that of the latter, indicating that the liposomal delivery system can effectively promote the gastrointestinal absorption of active ingredients from TCM. Tissue distribution experiments further confirmed that the concentrations of baicalin in the liver, kidney, and lung were 5.59, 2.33, and 1.25 times higher than those of the suspension, respectively, demonstrating favorable organ-targeting potential [52].

Moreover, polymeric micelles are core–shell nanoscale drug delivery systems formed by the spontaneous self-assembly of amphiphilic block copolymers in water. Their preparation methods include nanoprecipitation, dialysis, solvent evaporation, thin-film hydration, and direct dissolution. Among these, nanoprecipitation involves co-dissolving the polymer and drug in an organic solvent, followed by slow injection into an aqueous phase and subsequent removal of the organic solvent to induce self-assembly, which offers advantages such as simple operation and controllable particle size. The carriers of this system are mainly composed of hydrophilic blocks (e.g., methoxypolyethylene glycol) and hydrophobic blocks (e.g., polycaprolactone). The former prolongs the systemic circulation time and reduces immune recognition, while the latter encapsulates poorly soluble drugs into the core through hydrophobic interactions. The resulting polymeric micelles not only significantly improve the water solubility and stability of active ingredients from TCM, but also achieve sustained drug release and prolong the in vivo half-life, thus representing an important nanocarrier platform with broad application prospects in the field of TCM. For example, curcumin-loaded mPEG-PCL micelles prepared by nanoprecipitation increased the area under the plasma concentration-time curve, half-life, and peak plasma concentration 52.8, 4.63, and 7.51 times, respectively, compared with free curcumin, markedly improving the in vivo delivery efficiency and therapeutic benefits of the drug [53].

Metal–organic frameworks (MOFs) are novel porous nanocarriers with a periodic three-dimensional network structure, formed by connecting metal ions or metal clusters with organic linkers via coordination bonds. MOFs are primarily prepared using strategies such as the solvothermal method, hydrothermal method, microwave-assisted synthesis, and mechanochemical method. Among these, the solvothermal method is the most widely applied. It involves reacting metal salts with organic linkers in a closed system under high temperature and high pressure, allowing precise control over crystal morphology and pore size. In terms of carrier composition, the core structural units of MOFs include metal nodes and organic linkers, which form a highly ordered porous framework through coordination interactions. Benefiting from their ultra-high specific surface area, tunable pore size, and surface chemical modifiability, MOFs can efficiently encapsulate hydrophobic active ingredients of TCM, significantly improve the water solubility of these drugs, and achieve sustained and controlled release through gradual degradation of the framework. Moreover, due to their tunable pore structure, MOFs also enable tailored design of particle size and surface properties to accommodate different routes of administration. In a study on the delivery of curcumin using the Mg-MOF-74 series, in vitro release experiments performed with a drug loading of 30 wt% demonstrated that Mg-MOF-74 increased the release rate constant of curcumin from 0.15 h^−1^ to 0.30 h^−1^, achieving a two-fold enhancement in release rate, thereby effectively promoting drug dissolution and delivery efficiency [54].

The biomimetic modification strategy further expands the carriers’ functional dimensions. Biomimetically modified nanocarriers are intelligent drug delivery systems that directly “coat” naturally derived cell membranes onto the surfaces of synthetic nanocores. Their preparation process generally consists of three major steps: first, the nanocores loaded with TCM and its active ingredients are prepared using techniques such as solvent evaporation or nanoprecipitation; second, cell membranes are extracted and purified from macrophages; and third, the membranes and cores are integrated through gentle methods such as physical co-extrusion or ultrasonic fusion to form biomimetic nanoparticles with natural “camouflage”. The shells of biomimetically modified nanocarriers are derived from functionalized macrophage membranes, retaining key protein clusters on the membrane surfaces, which constitute the functional recognition interfaces of the biomimetic structure. The inner cores can be constructed using a variety of biocompatible materials. Benefiting from this structure, erythrocyte membrane-coated nanocarriers, leveraging proteins such as CD47, exhibit excellent immune evasion capabilities, significantly reducing their capture and clearance by macrophages in vivo, thereby prolonging the residence time of the drug in the blood circulation. Meanwhile, the macrophage-associated proteins retained on the surfaces of the nanocarriers enable the nanoparticles to naturally target inflammatory lesions or tumor tissues, achieving efficient active targeted delivery [55].

To sum up, the strategy of nanocarrier encapsulation systematically overcomes the inherent limitations of stability and targeted delivery of TCM and its active ingredients through the combination of multifunctional materials and fine structure design, which not only significantly improves the bioavailability and therapeutic efficacy of drugs, but also provides a precise and controllable delivery platform for reducing the toxicity of anti-tumor drugs, showing broad clinical translation prospects.

#### 4.1.3. Chemically Coupled Nanomedicines

Chemically coupled nanomedicines typically link active ingredients of TCM to amphiphilic polymers or functional ligands via covalent bonds, forming prodrug-type nano-delivery systems. Common preparation methods include esterification, amide condensation, and disulfide bond coupling, and the resulting products can self-assemble into micelles or nanoparticles in aqueous media. The carrier molecules mainly consist of hydrophilic and hydrophobic segments, wherein the hydrophobic segment encapsulates or directly conjugates the drug, while the hydrophilic segment prolongs systemic circulation time and reduces immune recognition, thereby conferring long-circulating properties. The chemical coupling bonds mainly include ester, amide, hydrazone, and disulfide bonds. Among these, ester bonds are hydrolytically cleaved by esterases in vivo, hydrazone bonds are cleaved in response to an acidic microenvironment, and disulfide bonds undergo reductive cleavage sensitive to high concentrations of intracellular glutathione. Therefore, through rational design of chemical bonds, such nanomedicines can specifically respond to microenvironmental stimuli at the diseased site, achieving controlled drug release and thus significantly increasing drug concentration at the target site while reducing off-target side effects. Taking the complex micelles self-assembled from a curcumin-glycyrrhetinic acid-disulfide bond prodrug and glycyrrhizic acid as an example, this chemical coupling strategy not only markedly improves the solubility of curcumin but also endows the system with liver-active targeting and glutathione-responsive release capabilities [56]. In in vitro release studies, the cumulative release rate reached 67.5% within 24 h under 10 mM glutathione, significantly higher than 40% in the absence of glutathione. Cellular uptake experiments showed that the fluorescence intensity of the micelle group was 1.8 times that of the free drug. In vivo distribution results demonstrated that the accumulation of these chemically coupled micelles in tumor tissue was 3.6 times that of the non-targeted control group, fully illustrating the advantages of the chemical coupling strategy in improving pharmacokinetic behavior and targeted delivery efficiency.

To sum up, chemically coupled nanomedicines not only overcome the problems of low solubility and rapid metabolism of TCM but also enhance the attenuation effect through multiple channels, and realize the targeted evasion and mechanism intervention of anti-tumor drug toxicity by precisely regulating the interaction between TCM and carriers, which provides a new strategic direction for anti-tumor treatment.

### 4.2. Targeting of Nanomedicines

Nanocarriers can accurately deliver the active ingredients of TCM to target organs via passive or active targeting, reducing drug distribution to non-target organs and thereby mitigating off-target toxicity. Passive targeting primarily relies on the enhanced permeability and retention (EPR) effect of tumor tissues. Because solid tumors possess an abnormally proliferating vascular system with large gaps between vascular endothelial cells (typically ranging from 100 to 800 nm) and impaired lymphatic drainage in the tumor region, nanoparticles within a specific size range can be specifically delivered to tumor tissues or inflammatory lesions, achieving selective drug accumulation. In the field of cardioprotection, Pluronic^®^ F-127 polymer micelles are used as nanocarriers, leveraging their nano-size to achieve the EPR effect, enabling passive targeting and enrichment at tumor sites. These micelles are loaded with active ingredients of TCM with cardioprotective effects, which not only significantly improve the solubility and stability of these hydrophobic ingredients but also enhance their tumor accumulation in ovarian cancer models through the EPR effect, thus cooperating with DOX to play an anti-tumor role. The experimental results demonstrated that this nanosystem not only effectively mitigated the cardiac toxicity induced by DOX, as evidenced by the reduced decline in left ventricular ejection fraction (LVEF) and decreased cardiac troponin I (cTnI) release, but also decreased the required DOX dosage through chemosensitization. Ultimately, it achieved the goal of an integrated TCM and modern medicine treatment approach by toxicity attenuation and efficacy potentiation [57]. However, passive targeting strategies face significant challenges in clinical translation. The EPR effect exhibits high heterogeneity not only across different tumor types but also among different patients with the same tumor type; furthermore, factors such as variations in tumor vascular density, elevated interstitial fluid pressure, and stromal barriers frequently restrict the effective penetration and retention of drugs. In addition, the non-specific distribution of nanoparticles in vivo may still lead to their massive accumulation in macrophage-rich organs such as the liver and spleen. It is precisely due to these inherent limitations of passive targeting in tumor-specific recognition and tissue penetration depth that active targeting strategies have emerged, which endow nanocarriers with the ability to precisely recognize diseased cells through functional modifications.

**Figure 3:**
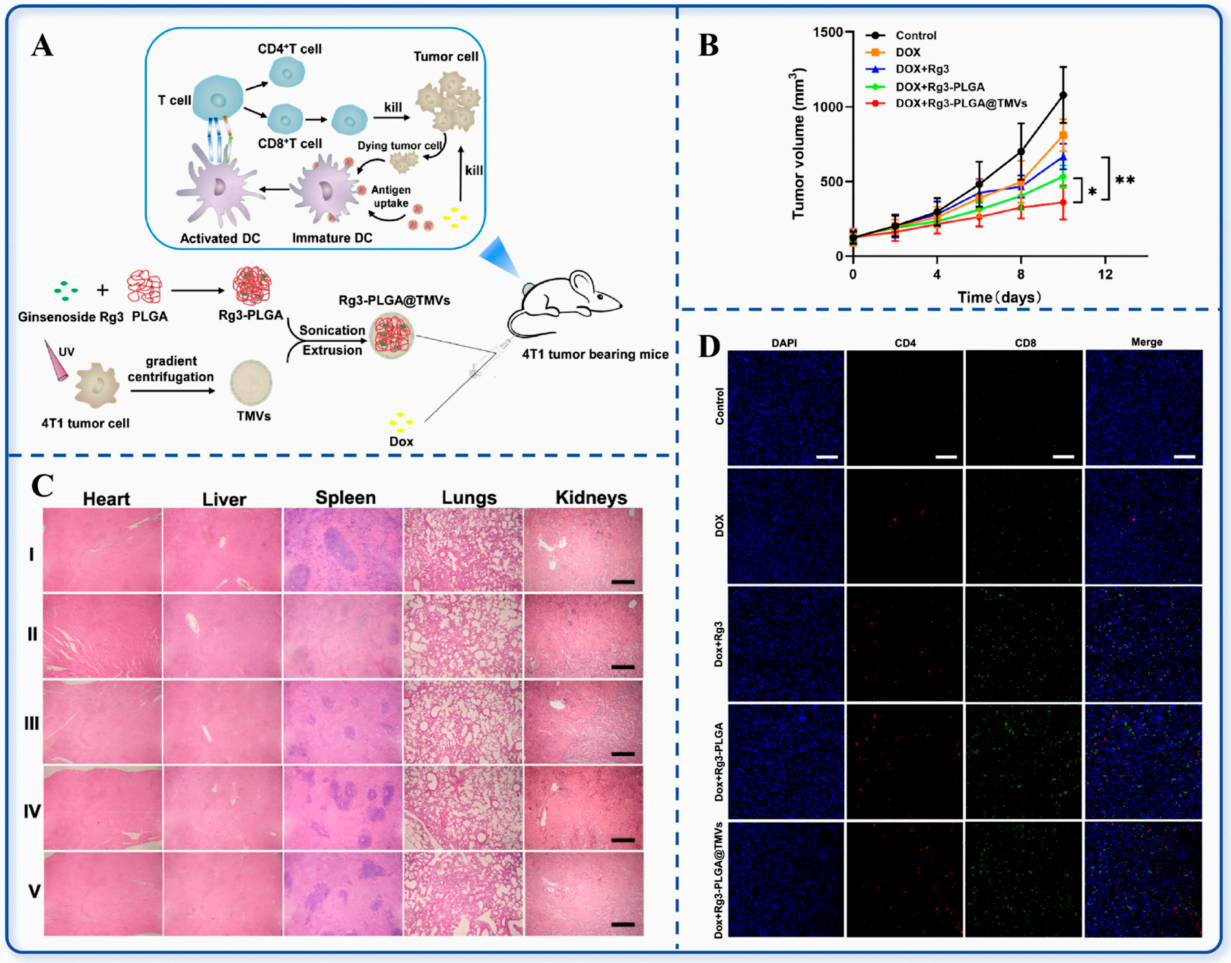
(**A**) Schematic diagram of the preparation process and anti-tumor mechanism of Rg3-PLGA@TMVs nanosystem. (**B**) Tumor growth curves. (* 0.01 < *p* < 0.05, ** 0.001 < *p* < 0.01). (**C**) H&E staining showing the pathological changes in the main organs (I: control; II: DOX; III: DOX +Rg3; IV: DOX + Rg3-PLGA; V: DOX + Rg3-PLGA@TMVs. Scale bar: 100 μm). (**D**) Immunofluorescence analysis showing the infiltration of CD4+ (red) and CD8+ (green) T cells in tumor tissues (scale bar: 100 μm). Copyright 2024 Biomaterials Science [58].

Active targeting strategies involve conjugating specific ligands (such as antibodies, peptides, aptamers, or small molecules) to the surface of nanocarriers, enabling them to recognize and bind to overexpressed receptors on the surface of target organs or cells, thereby achieving precise drug delivery and enhancing cellular uptake efficiency. By specifically recognizing certain receptors, these targeting ligands mediate the entry of nanomedicines into target cells via receptor-mediated endocytosis pathways, which not only increases the enrichment concentration of drugs at the target site but also effectively reduces drug accumulation in non-target tissues. For example, Zhang et al. discussed how to overcome the problems of poor water solubility and low bioavailability of ginsenoside Rg3 using nanotechnology, and used tumor cell-derived microvesicles (TMVs) to enhance its targeting and immunomodulatory capabilities. The core principle of the study is that ginsenoside Rg3 is encapsulated in PLGA nanoparticles and then coated with TMV membranes to form Rg3-PLGA@TMVs, thereby enabling controlled release and homologous targeting (Figure 3A). Among the key results, Figure 3B shows that, compared with DOX monotherapy, DOX combined with Rg3-PLGA@TMVs significantly reduces tumor weight, indicating strong tumor growth inhibition. Histological analysis in Figure 3C further confirms the safety of this strategy: the DOX monotherapy group exhibited cardiotoxic lesions such as loose myocardial cell arrangement, whereas the DOX+Rg3-PLGA@TMVs group showed no abnormalities in cardiac tissue, suggesting that the nanomedicine can effectively alleviate the organ toxicity of DOX. Figure 3D confirms enhanced infiltration of CD4+ and CD8+ T cells via immunofluorescence, indicating the promotion of an anti-tumor immune response. In vivo distribution studies showed that the fluorescence intensity of PLGA@TMVs at the tumor site was significantly higher than that of unmodified PLGA nanoparticles, indicating that the microvesicle coating endows nanoparticles with homologous targeting capability. Compared with free DOX, the combination with Rg3-PLGA@TMVs greatly increased the tumor inhibition rate from 24.98% to 66.61%, with no significant body weight loss in mice, no pathological changes in cardiac tissue, and a return of white blood cell count to normal levels. Serum creatinine, urea, transaminases, and other liver and kidney function indicators also showed no significant abnormalities. These targeted delivery advantages effectively alleviated the cardiac and systemic toxicity of DOX, providing a feasible strategy for the safe enhancement of chemotherapy drug efficacy. To sum up, this strategy skillfully leverages the homologous targeting and antigen-presenting functions of TMVs to achieve dual optimization of drug delivery and immune regulation. However, its limitations may lie in the standardized large-scale preparation of TMVs and the need for further long-term in vivo safety verification [58].

### 4.3. Environmental Response of Nanomedicines

Stimuli-responsive drug delivery systems can trigger the controlled release of drugs under specific endogenous or exogenous stimuli, a characteristic that holds significant value for precise toxicity-reducing therapies. Endogenous responsiveness typically utilizes specific physiological signals within the pathological microenvironment, such as elevated ROS levels, acidic pH, increased glutathione concentrations, or the overexpression of specific enzymes. Conversely, exogenous responsiveness relies on externally applied physical stimuli, such as light, magnetic fields, and ultrasound. When developing endogenous response systems, researchers typically exploit differences in the microenvironment between diseased and healthy tissues to design nanocarriers that remain stable under normal physiological conditions but undergo structural changes or chemical bond breakage in response to specific pathological signals. In contrast, exogenous response systems enable spatially and temporally controlled drug release through the application of external physical stimuli. In these systems, nanocarriers can convert forms of energy—such as light or magnetic fields—into heat or reactive oxygen species, thereby combining the dual functions of drug delivery and physical therapy. Combining these smart controlled-release properties with multi-target TCM and its active ingredients allows the nanomedicine to remain stable in normal tissues—thereby reducing off-target exposure—while enabling the rapid and efficient release of TCM in damaged areas upon response to specific stimulus signals. This approach alleviates the toxicity to normal organs caused by anti-tumor drugs and, through synergistic effects, enhances the overall anti-tumor efficacy, providing a new strategy for developing novel nanomedicines that “toxicity attenuation and efficacy potentiation”.

#### 4.3.1. Endogenous Response of Nanomedicines

**Figure 4:**
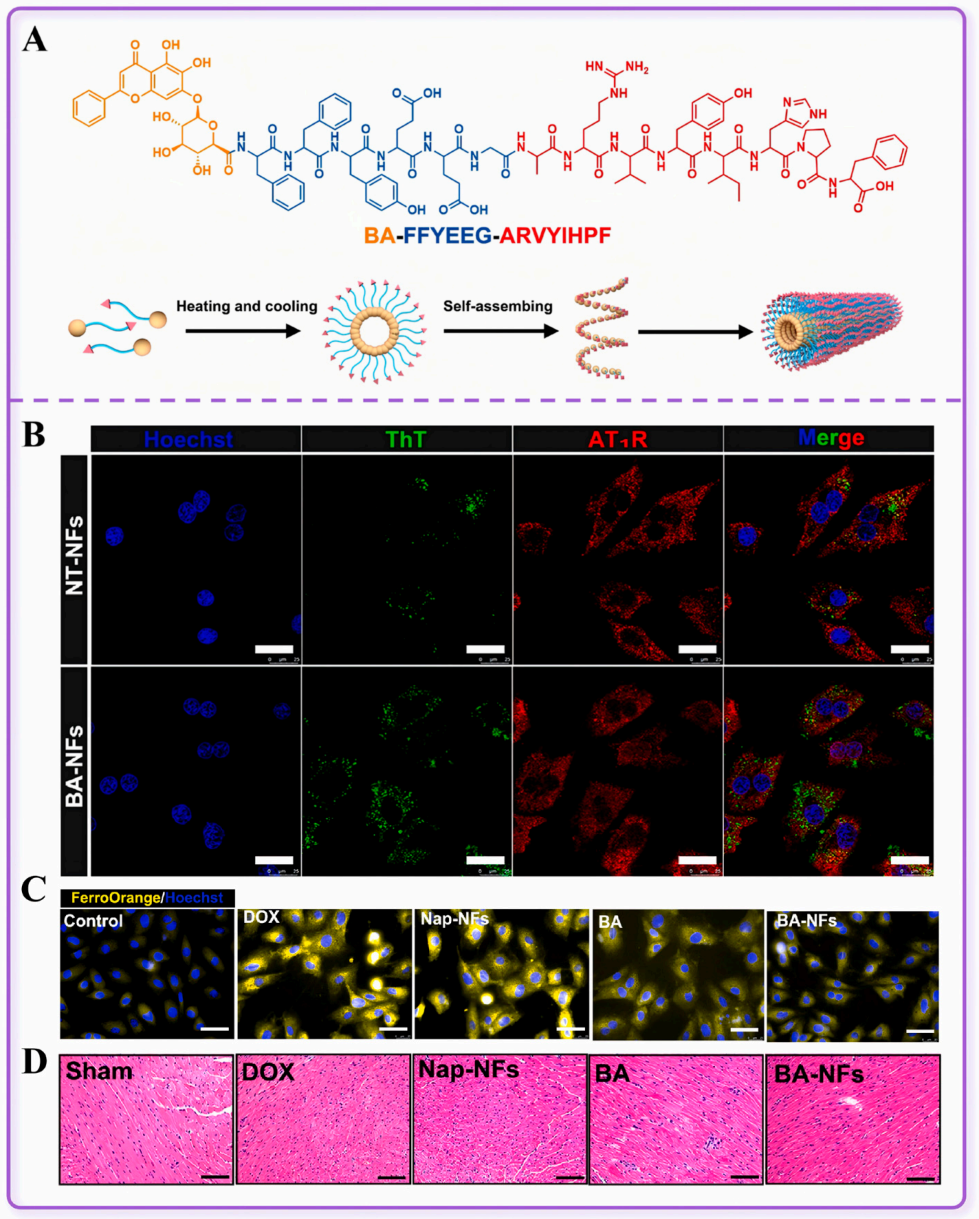
(**A**) The process involves the self-assembly of BA-NFs, which is facilitated by the combination of baicalin (BA), self-assembling peptide FF (FFYEEG), and targeting peptide (ARVYIHPF), under the influence of heating and cooling. (**B**) The targeting ability of BA-NFs to bind to angiotensin II type 1 receptors (AT1R) in H9c2 cells by ThT staining (scale bar: 25 μm). (**C**) Intracellular Fe^2+^ level (scale bar: 100 μm). (**D**) The representative H&E staining images showed the pathological changes in heart tissue in different groups of mice (scale bar: 100 μm). Copyright 2024 Journal of Controlled Release [51].

Endogenous responsive nanocarriers achieve targeted drug delivery by sensing microenvironmental signals specific to diseased tissues. Among these, ROS-responsive strategies have attracted particular attention for their ability to mitigate damage to normal organs caused by anti-tumor drugs. When metabolically active renal tubules or myocardial tissues are damaged by anti-tumor drugs, high levels of ROS are produced locally, further aggravating tissue damage. To solve this problem, researchers designed ROS-responsive nanoparticles to load and deliver the active ingredients of TCM with antioxidant effects. These nanoparticles remain stable in normal tissues; however, once they reach the damaged sites, high ROS levels trigger structural changes in the nanoparticles, thereby enabling the controlled release of TCM at specific points. For example, during treatment of CDDP, the kidneys are easily damaged. A ROS-responsive metal-polyphenol nanoenzyme can specifically accumulate in the kidney and trigger the release of baicalein (Ba) carried by it in a high-ROS environment, synergistically enhancing the ROS scavenging ability of the nanosystem and protecting renal tubular epithelial cells in multiple ways, thus alleviating the nephrotoxicity of CDDP [59]. In addition, ROS-responsive nanocarriers can also be used to alleviate the cardiotoxicity caused by anthracycline drugs such as DOX. Zeng et al. discussed the potential of baicalin-peptide supramolecular self-assembled nanofibers (BA-NFs) to alleviate DOX-induced cardiomyopathy by inhibiting ferroptosis. Based on the common myocardial toxicity in DOX chemotherapy, this study proposed a targeted delivery strategy: BA-NFs are composed of baicalin and the targeting peptide ARVYIHPF through a self-assembly sequence, which can specifically target the upregulated AT1R in cardiomyocytes, thus accurately delivering drugs and inhibiting the ferroptosis pathway (Figure 4A) [51]. In this study, the self-assembled nanofibers (BA-NFs) exhibited a critical aggregation concentration of 68.94 μM in vitro and achieved over 98% release of baicalin within 20 h, effectively addressing the poor solubility and low bioavailability of the active ingredients of TCM. In terms of targeting ability, Figure 4B confirms via ThT staining that BA-NFs can efficiently bind to damaged cardiomyocytes, and Figure 4C further reveals the inhibitory effect of BA-NFs on ferroptosis. In vivo verification, the histological results in Figure 4D directly demonstrated the myocardial protective effect of BA-NFs: the interstitial spaces in the hearts of DOX-treated mice were enlarged and vacuolated, whereas the BA-NFs group significantly alleviated these pathological changes, with the myocardial structure being closer to that of the normal group. To sum up, the nanomedicine BA-NFs provides a novel therapeutic strategy for DOX-induced myocardial toxicity through targeted delivery and inhibition of ferroptosis. However, BA-NFs may cause a slight increase in blood pressure, and their long-term safety needs further evaluation. In the future, the peptide sequence can be optimized to reduce its potential risk.

#### 4.3.2. Exogenous Response of Nanomedicines

Unlike endogenous responsive nanocarriers, which depend on the characteristics of the tumor microenvironment, exogenous responsive nanocarriers achieve spatiotemporally controlled drug release through the application of external physical stimuli, thereby enabling researchers to actively regulate drug release kinetics. It is worth noting that, exogenous responsive nanocarriers can not only serve as delivery tools but also integrate multimodal therapeutic functions, such as photothermal and photodynamic therapies, through their own design, forming a synergistic mechanism with the loaded active ingredients of TCM to enhance the toxicity-reducing effect. Photothermal therapy utilizes nanomaterials to convert light energy into thermal energy under near-infrared irradiation, and kills tumor cells through localized thermal effects. Photodynamic therapy, on the other hand, relies on photosensitizers to generate ROS upon excitation by a specific wavelength of light, thereby inducing cellular oxidative damage and apoptosis. Both physical therapy modalities offer advantages such as high spatiotemporal controllability, minimal invasiveness, and low drug resistance. However, a single modality often faces challenges such as limited tissue penetration depth or restrictions imposed by the hypoxic microenvironment. Combining physical therapy with the delivery of TCM and its active ingredients can exert multiple synergistic effects—integrating physical destruction with the regulation of oxidative stress, inflammatory responses, and the immune microenvironment by TCM and its active ingredients—thereby achieving the integrated therapeutic goal of “toxicity attenuation and efficacy potentiation”.

**Figure 5:**
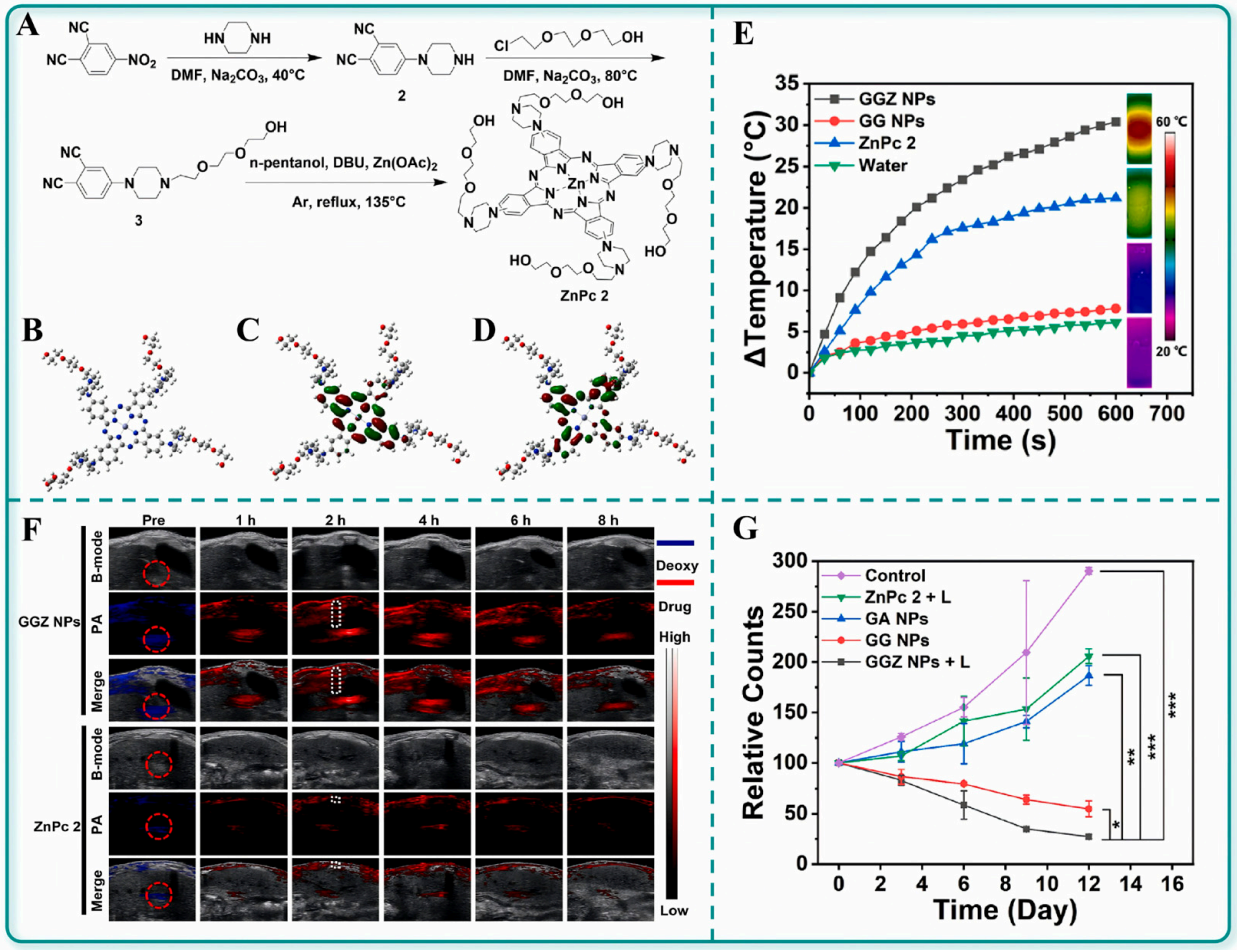
(**A**) The synthetic route of the ZnPc2 photosensitizer. (**B**–**D**) Highest occupied molecular orbital (HOMO), and lowest unoccupied molecular orbital (LUMO) visualization, to clarify the electronic structure characteristics. (**E**) Photothermal performance curve of GGZ NPs. (**F**) Photoacoustic imaging in vivo, showing the accumulation and distribution of GGZ NPs in the tumor site with time. (The red dotted area represents the drug distribution, while the white dotted area represents the penetration depth of the nanoparticles). (**G**) The quantitative analysis of signal intensity of tumor-related bioluminescence imaging between treatment groups. (* *p* < 0.05, ** *p* < 0.01, *** *p* < 0.001, n = 3). Copyright 2025 Materials Today [61].

For example, in the study of ginsenoside Rg3, Pluronic F127 micelles are used as nanocarriers to load ginsenoside Rg3 via self-assembly, which not only improves its water solubility and oral bioavailability but also endows the system with photothermal conversion ability. When used in combination with DOX, the nanocarriers produce thermal effects under illumination to directly kill tumor cells, while ginsenoside Rg3 alleviates DOX-induced cardiotoxicity through antioxidant and anti-inflammatory effects, thereby achieving synergy [60]. Similarly, the strategy of carrier-free nanoparticles shows the potential of natural product self-assembly. Wang et al. studied carrier-free nanoparticles (GG NPs) based on the natural compound gambogic acid (GA) and glycyrrhizic acid (GL), and self-assembled them with the photosensitizer ZnPc2 to form GGZ NPs to enhance phototherapy in liver cancer. In this study, GG NPs were constructed using a molecular self-assembly strategy, which realized an internal “toxicity attenuation and efficacy potentiation” mechanism, where GA played an anti-tumor role while GL provided liver targeting and reduced toxicity. After the further introduction of the photosensitizer, GGZ NPs combined photothermal therapy (PTT) and photodynamic therapy (PDT) to enhance the therapeutic effect by disrupting the intracellular redox balance and inhibiting heat shock proteins (Figure 5A–D) [61]. As shown by the comparison of temperature curves in Figure 5E, under photothermal imaging, GGZ NPs raised the tumor temperature to 43 °C within 5 min—significantly higher than the 39 °C observed in the free ZnPc2 group—confirming their excellent photothermal performance. The photoacoustic imaging results in Figure 5F show that GGZ NPs exhibit active, targeted accumulation at tumor sites, thereby enhancing drug enrichment in liver cancer tissues. Further, the quantitative bioluminescence imaging analysis in Figure 5G shows that GGZ NPs exhibit a photothermal conversion efficiency as high as 80.8%. Under acidic conditions, their singlet oxygen yield increases two-fold. Following dual-wavelength laser irradiation, the tumor growth inhibition rate is 1.8 times higher than that of ZnPc-2. In addition, GG NPs effectively reduced the toxicity of the natural compound garcinic acid to normal liver cells, resulting in a significant decrease in aspartate aminotransferase and alanine aminotransferase levels.

In recent years, the design of multifunctional nanoplatforms has evolved from single exogenous responses to synergistic multi-response systems. For instance, nanocarriers possessing a dual-responsive mechanism to both endogenous pH and exogenous photothermal stimuli can achieve targeted drug release more precisely within complex pathological microenvironments. In the acidic tumor microenvironment, chitosan molecules on the surface of liposomes undergo conformational changes due to the protonation of amino groups, which increases the permeability of the lipid bilayer and triggers drug release. Simultaneously, under external near-infrared light irradiation, gold nanoshells efficiently convert light energy into thermal energy through the plasmon resonance effect. The resulting local high temperature not only induces a gel-to-liquid crystalline phase transition in the lipid membrane to accelerate drug release, but also enhances cellular uptake of the drug via thermal effects [62]. Overall, nanocarriers, by integrating multimodal therapeutic functions, synergize with ingredients of TCM at the molecular level, thereby improving the precision of tumor treatment and achieving the dual goals of toxicity attenuation and efficacy potentiation through complementary mechanisms, providing new insights for clinical combined medication.

### 4.4. Challenges and Strategies for Nanomedicines in Alleviating the Toxicity of Anti-Tumor Drugs

As an innovative strategy to alleviate the toxicity of anti-tumor drugs, nanomedicines show significant potential by improving the delivery efficiency and targeting of TCM. However, their clinical translation faces systematic challenges: in terms of safety, the biocompatibility of carriers is insufficient, long-term toxicity data are lacking, and exogenous materials may trigger immune responses or cause organ accumulation and damage [63]. In terms of therapeutic precision, the heterogeneity of the tumor microenvironment and physiological barriers affect targeting efficiency, which can easily lead to off-target accumulation and toxicity, especially in multi-organ co-toxicity scenarios [64]. At the industrial level, there are problems with large-scale production, such as complex processes and difficult quality control. Batch-to-batch variations in particle size distribution and drug loading are significant, making it difficult for existing quality control standards to provide comprehensive coverage [65]. Among these, the lack of batch-to-batch reproducibility is one of the core bottlenecks hindering the clinical translation of nanomedicines. The synthesis process of nanocarriers is highly process-dependent. If each step is not strictly controlled, it will lead to significant differences in physicochemical properties and biological performance between batches, subsequently affecting modification efficiency and therapeutic effects. Meanwhile, nanomedicines face severe colloidal stability challenges during storage. Their high surface free energy makes them prone to aggregation and fusion, leading to drug leakage and a decline in efficacy. Although lyophilization is commonly used to improve long-term stability, the issue of variability among particles and components in lyophilized samples is prominent. Ice crystal formation during freezing, crystallization of lyoprotectants, and improper control of the glass transition temperature can all cause particle damage or aggregation after reconstitution, affecting the quality consistency of the product.

More complexly, this field also faces numerous intersecting challenges. At the formulation level, there is a lack of standardized guidelines for the selection of lyoprotectants; optimal lyophilization parameters vary drastically among different types of nanoparticles, and the scale-up effects of lyophilization process parameters during the transition from laboratory scale to industrial-scale batch production are difficult to control precisely. At the quality control level, due to the lack of unified standards for testing key quality attributes of nanomedicines, such as particle size, surface charge, and drug loading capacity, existing pharmaceutical regulatory laws are inadequate to fully cover the particularities of TCM nanopreparations. At the social and regulatory levels, significant individual differences among patients affect pharmacokinetic behaviors [66,67]. The definitions and evaluation criteria for TCM-based nanomedicines in relevant laws and regulations remain unclear. Furthermore, high production and commercialization costs restrict clinical accessibility [68].

To overcome these bottlenecks, the research and development mindset must shift from laboratory exploration to full-chain quality control. Addressing the issue of colloidal stability, optimizing formulation design is a foundational strategy. Steric hindrance effects can be enhanced by screening suitable surfactants or polymers to inhibit aggregation phenomena [69]. Regarding the control of lyophilization variability, the rational selection of lyoprotectants (such as disaccharides like trehalose and sucrose) is crucial. Simultaneously, regulating the annealing steps, optimizing the lyophilization cycle, and precisely controlling the glass transition temperature can significantly improve the integrity and reconstitution rate of the lyophilization [70]. To solve the difficult problem of batch-to-batch reproducibility, microfluidic technology and continuous manufacturing processes can achieve precise control over reaction conditions, effectively reducing batch-to-batch variation [71]. On this basis, it is necessary to develop degradable intelligent responsive carriers in the future [72], implement continuous and modular production, establish a multidimensional quality control system, and construct a multilevel targeted and stimulus-responsive delivery system to enhance precision. Simultaneously, interdisciplinary collaboration should be strengthened to improve the regulatory framework, promote individualized treatment, and develop low-cost technologies [73]. In terms of lyophilization process optimization, systematic design of lyoprotectant screening and process parameters should be conducted for different types of nanomedicines, and artificial intelligence-assisted lyophilization cycle optimization strategies should be explored to improve the quality and production efficiency of lyophilized products. At the regulatory level, there is an urgent need to formulate exclusive guiding principles and technical specifications for TCM nanopreparations, establish unified evaluation standards for physicochemical properties and biological effects, and encourage large-scale, multi-center preclinical and clinical studies to accumulate sufficient efficacy and safety data. Regarding cost and accessibility, digital twin frameworks and big data analytics can be leveraged to optimize the supply chain and quality management systems, while low-cost, high-throughput preparation processes should be explored to lower the threshold for commercialization. Although the nanocarrier systems of TCM have great potential in enhancing bioavailability [74], their overall clinical translation still faces severe challenges from theory to practice [75]. Only by systematically addressing the above challenges can we facilitate a substantial leap from basic research to clinical application in this field.

## 5. Conclusions and Prospects

This review systematically summarizes and discusses the research progress on the mechanisms of toxicity of anti-tumor drugs across three aspects: nonspecific cell damage from chemotherapy drugs, off-target effects and endothelial damage from targeted drugs, and irAEs from immunotherapy drugs. This review also focuses on the multi-target regulatory potential of active ingredients of TCM in alleviating the above toxicity. However, common problems with ingredients, such as poor water solubility and low bioavailability, limit their clinical application. In recent years, the introduction of nanotechnology has provided a new solution to the efficient delivery of TCM. By constructing different forms of nanomedicines, the solubility, stability, and targeting properties of TCM can be effectively improved. Furthermore, with the help of organ-specific enrichment and stimuli-responsive release strategies, the therapeutic efficacy can be enhanced and systemic toxicity can be reduced.

However, the existing clinical problems still cannot be ignored. First of all, there is still a lack of systematic intervention strategies for the multi-organ co-toxicity of anti-tumor drugs (such as nephrotoxicity and neurotoxicity caused by CDDP). Secondly, although TCM and its active ingredients possess the advantage of multi-target regulation, they also inherently exhibit potential organ toxicity. These toxicities often coexist with the therapeutic effects of the active ingredients, resulting in a narrow therapeutic window, posing safety hazards to clinical applications. In addition, the clinical application of TCM nanocarriers still faces technical bottlenecks in biocompatibility, long-term safety, and large-scale preparation. Meanwhile, the upgrade to multimodal intelligent response systems, the synergistic detoxification of combined TCM and modern medicine treatment strategies, and the multi-component nano-design strategy based on TCM theory still need further exploration.

In the future, the management of anti-tumor drug toxicity needs to advance in the following directions based on multidisciplinary collaboration: first, developing multimodal activation systems capable of simultaneously responding to multiple biological signals to achieve more precise spatiotemporal intervention; second, exploring combined treatment strategies of TCM and modern medicine, enhancing anti-tumor efficacy and reducing toxicity through the synergistic action of natural compounds and anti-tumor drugs; third, introducing multi-component nanotechnology design strategies guided by TCM theory, achieving “one substance, multiple uses” of active components of TCM in nanomedicines. To address the organ toxicity issues inherent in TCM and its active ingredients, future advancements in nanotechnology should prioritize the research and development of novel nanomedicines such as nanocrystals and prodrug nanoassemblies, aiming to reduce direct organ exposure of active ingredients through physical encapsulation or chemical modification. Simultaneously, the development of combined nanodelivery systems based on TCM compatibility theory is essential, where toxic ingredients are co-loaded with compatibility ingredients with attenuation effects onto nanocarriers to achieve synergistic effects. Furthermore, it is imperative to establish a long-term toxicity evaluation system and standardized quality control methods for TCM nanomedicine systems, ensuring the safety of their clinical translation and application. In summary, through the deep integration of nanotechnology and TCM, it is expected to promote the management of anti-tumor drug toxicity towards precision, systematization, and intelligence, opening up new paths for cancer treatment.

## Figures and Tables

**Table 1:** Toxic mechanisms of anti-tumor drugs and detoxification mechanisms and effects of TCM and its active ingredients.

Drug Category	Typical Drug	Toxicity Type	Toxicity Mechanism	Active Ingredients/Formulas of TCM	Detoxification Mechanism	Detoxification Effect	Ref.
Chemotherapy drugs	Doxorubicin(DOX)	Cardiotoxicity	Disruption of reactive oxygen species (ROS)/DNA topoisomerase II beta and inhibition of AMPK pathway	Curcumin	Activation of PI3K/Akt/mTOR pathway and inhibition of NLRP3 inflammasome	Alleviation of cardiomyocyte pyroptosis and improvement of cardiac function	[23]
				Silymarin	Promotion of antioxidant, anti-inflammatory, and anti-apoptotic effects	Improvement of cardiac ejection fraction and reduction in myocardial enzymes and cardiac troponin (cTnI/cTnT) levels	[24]
	Cisplatin (CDDP)	Nephrotoxicity	Induction of excessive ROS production and inhibition of Nrf2/HO-1 antioxidant pathway	Quercetin	Inhibition of OCT2 function and activation of Nrf2/ARE pathway	Reduction in cisplatin accumulation in renal tubules and enhancement of antioxidant enzyme activity	[25]
	Doxorubicin		Inhibition of nephrin (key protein of podocyte slit diaphragm) expression and activation of NF-κB signaling pathway	Huaiqihuang granules	Enhancement of nephrin expression and inhibition of NF-κB pathway	Alleviation of renal podocyte damage	[26]
	Carboplatin	Myelosuppression	Inhibition of hematopoietic stem cell proliferation and differentiation, and reduction in megakaryocyte progenitor reactivity to thrombopoietin (TPO) in bone marrow microenvironment	Aqueous extract of carica papaya leaf	Upregulation of Interleukin-11 and TPO expression	Alleviation of thrombocytopenia	[27]
	5-Fluorouracil		Induction of oxidative stress	Ganoderma lucidum polysaccharide	Regulation of gut microbiota-immune axis, promotion of SCFAs production, and promotion of G-CSF expression	Enhancement of hematopoietic progenitor cell survival and differentiation	[28]
	5-Fluorouracil	Gastrointestinal toxicity	Activation of IL-6/STAT3 pathway and upregulation of inflammatory factors	Curcumin	Inhibition of IL-6/STAT3 pathway and reduction in pro-inflammatory cytokines	Restoration of intestinal mucosal barrier	[29]
	Irinotecan		Activation of TLR4/NF-κB pathway and promotion of inflammatory factor release	Ginsenoside Rh4	Enrichment of beneficial bacteria and inhibition of TLR4/NF-κB pathway	Restoration of tight junction proteins and alleviation of gastrointestinal mucositis	[30]
				Hesperidin	Upregulation of tight junction protein expression	Reinforcement of intercellular junction structures in intestinal epithelial cells	[31]
	Oxaliplatin		Induction of intestinal inflammatory factors and upregulation of TLR4/MyD88/NF-κB pathway	Jianpi Bushen formula	Inhibition of Gasdermin protein-mediated pyroptosis and reduction in downstream inflammatory cytokine release	Alleviation of nausea and vomiting, and improvement of chemotherapy completion rate	[32]
	Capecitabine						
Targeted drugs	Trastuzumab	Reversible cardiomyopathy	Inhibition of the NRG1/ErbB4-PI3K/Akt signaling and imbalance of the BCL-xL/Bax ratio	Salvianolic acid B	Inhibition of ROS-mediated endothelial damage and restoration of eNOS/cGMP pathway	Improvement of vasodilation and tissue perfusion	[33]
	Bevacizumab	Hypertension and thrombosis	Reduction in endothelial nitric oxide synthases and elevation of endothelin-1 and plasminogen activator inhibitor-1	Ellagic acid	Promotion of antioxidant effects, mitigation of mitochondrial oxidative stress, and restoration of electron transport chain	Improvement of left ventricular dysfunction	[34]
	Gefitinib	Hepatotoxicity	Activation of p53/p21 pathway and induction of hepatocyte cell cycle arrest	Glycyrrhizic acid	Regulation of p53/p21 pathway	Alleviation of hepatocyte cell cycle arrest and protection of liver function	[35]
	Dasatinib	Gastrointestinal toxicity	Activation of ROCK kinase and promotion of myosin light chain (MLC) phosphorylation	Ruscogenin	Activation of ErbB4/YAP pathway and inhibition of ROCK/MLC pathway	Stabilization of intestinal vascular endothelial barrier and reduction in permeability	[36]
				Yiqi Fumai formula	Inhibition of ROCK/MLC pathway and upregulation of tight junction proteins	Alleviation of intestinal leakage	[37]
	Cetuximab		Blockade of EGFR/VEGF pathway	Huangci granules	Activation of synergistic mechanisms and regulation of multiple targets	Prolongation of progression-free survival (PFS) and reduction in adverse events such as leukopenia, fatigue, and hypertension	[38]
	Bevacizumab						
	Gefitinib	Skin toxicity	Upregulation of pro-inflammatory factors and inhibition of keratinocyte differentiation	Honeysuckle	Inhibition of NF-κB/MAPK pathway and inhibition of inflammatory factor production and release	Reduction in rash incidence and severity, and shortening of pruritus relief time	[39]
	Regorafenib		Inhibition of VEGFR1-3 and PDGFR-β	Shouzuping formula	Regulation of VEGF pathway, inhibition of inflammatory factors, and improvement of microcirculation	Alleviation of hand-foot skin reaction	[40]
Immunotherapy drugs	PD-1/PD-L1 inhibitors	irAEs	Inhibition of immunosuppressive function of regulatory T cells and upregulation of pro-inflammatory factor expression	Gegen Qinlian tablets	Regulation of IL-6 signaling pathway and inhibition of NLRP3 inflammasome-mediated pyroptosis	Reduction in irAEs incidence and delay of onset time	[41]
	PD-1 inhibitors	T-cell exhaustion-related toxicity	Upregulation of TIM-3 expression and impairment of CD8+ T cell effector function	Curcumin	Downregulation of PD-L1, PD-L2, and Galectin-9, and blockade of PD-1/TIM-3 signaling pathway	Restoration of CD8+ T cell function	[42]
	CTLA-4 inhibitors	Immune-mediated colitis	Necrosis of epithelium induced by tumor necrosis factor-α (TNF-α)	Kushen formula, Scutellaria baicalensis, Epimedium	Regulation of Th17 and Treg cell balance, inhibition of IL-6/IL-17, and elevation of IL-10/TGF-β	Restoration of intestinal barrier, regulation of microbiota, and alleviation of inflammation	[43]
	PD-1 inhibitors			Atractylenolide II	Inhibition of NF-κB p65/PD-L1 pathway and enhancement of CD8+ T cell infiltration	Enhancement of synergistic anti-tumor effects and reduction in immune-related colitis incidence	[44]

## Data Availability

Data will be made available on request.

## Abbreviations

The following abbreviations are used in this manuscript:

5-FU	5-Fluorouracil
ADP	Adenosine diphosphate
Akt	Protein kinase B
ALK	Anaplasticlymphoma kinase
AMPK	AMP-activated protein kinase
ARE	Antioxidant response elements
ASK1	Apoptosis signal-regulating kinase 1
AT1R	Angiotensin II type 1 receptor
ATM	Ataxia telangiectasia mutated
Bax	BCL-2-associated X protein
BCL-2	B-cell lymphoma-2
BCL-xL	B-cell lymphoma-extra large
BTK	Bruton’s tyrosine kinase
CAR-T	Chimeric antigen receptor T-cell immunotherapy
CDDP	Cisplatin
CDK4	Cyclin dependent kinase 4
CDK6	Cyclin dependent kinase 6
cGMP	Cyclic guanosine monophosphate
Chk2	Checkpoint kinase 2
CK	Creatine kinase
COX-2	Cyclooxygenase-2
CTLA-4	Cytotoxic T-lymphocyte-associated protein 4
cTnT	Cardiac troponin T
DOX	Doxorubicin
EEG	Electrocardiogram
EF	Ejection fraction
EGFR	Epidermal growth factor receptor
eNOS	Endothelial nitric oxide synthase
ErbB4	Erb-B2 receptor tyrosine kinase 4
ERK	Extracellular regulated protein kinase
FoxO3a	Forkhead box O3
FS	Fractional shortening
GPX4	Glutathione peroxidase 4
GSH	Glutathione
GSSG	Oxidized glutathione
HER2	Human epidermal growth factor receptor 2
HO-1	Heme oxygenase-1
IL-6	Interleukin-6
IL-10	Interleukin-10
IL-11	Interleukin-11
IL-17	Interleukin-17
JNK	C-Jun N-terminal kinase
LDH	Lactate dehydrogenas
MAPK	Mitogen-activated protein kinase
MET	Mesenchymal–epithelial transition factor
MLC	Myosin light chain
MRI	Magnetic resonance imaging
MTOR	Mammalian target of rapamycin
MTX	Methotrexate
MyD88	Myeloid differentiation primary response 88
NF-κB	Nuclear factor kappa-B
NLRP3	Nucleotide-binding oligomerization domain-like receptor protein 3
NQO1	NAD(P)H:quinone oxidoreductase 1
Nrf2	Nuclear factor erythroid 2-related factor 2
NRG1	Neuregulin 1
OCT2	Organic cation transporter 2
p21	Cyclin-dependent kinase inhibitor 1A
p53	Tumor protein 53
PARP	Poly ADP-ribose polymerase
PD-1	Programmed cell death-1
PD-L1	Programmed cell death ligand 1
PD-L2	Programmed cell death ligand 2
PGC-1α	Peroxisome proliferator-activated receptor γ coactivator 1-alpha
PI3K	Phosphatidylinositol-3 kinase
ROCK	Rho-associated protein kinase
ROS	Reactive oxygen species
SIRT1	Silent information regulator 1
Smad	Smad protein
SN-38	7-Ethyl-10-hydroxycamptothecin
STAT3	Signal transducer and activator of transcription 3
T2WI	T2-weighted imaging
TCM	Traditional Chinese medicine
TGF-β	Transforming growth factor-β1
Th17	T helper cell 17
ThT	Thioflavin T
TIM-3	T-cell immunoglobulin and mucin-domain containing molecule 3
TKI	Tyrosine kinase inhibitors
TLR4	Toll-like receptor 4
TNF	Tumor necrosis factor
TNF-α	Tumor necrosis factor-α
Treg	Regulatory T cells
VEGF	Vascular endothelial growth factor
VEGFR	Vascular endothelial growth factor receptor
YAP	YES-associated protein
